# Prevalence of Preoperative Anxiety and Its Relationship with Postoperative Pain in Foot Nail Surgery: A Cross-Sectional Study

**DOI:** 10.3390/ijerph17124481

**Published:** 2020-06-22

**Authors:** Daniel Navarro-Gastón, Pedro V. Munuera-Martínez

**Affiliations:** Department of Podiatry, University of Seville, 41009 Seville, Spain; daniel.navarro.gaston@gmail.com

**Keywords:** anxiety, APAIS, foot, nail, surgery, minor surgical procedures, postoperative pain

## Abstract

Preoperative anxiety has been studied in different medical disciplines, but it is unknown in minor surgical procedures such as foot nail surgery. This study aimed to determine the prevalence of preoperative anxiety and postoperative pain in foot nail surgery. The validated Amsterdam preoperative anxiety and information scale (APAIS) was used to evaluate preoperative anxiety and the need for information in 155 patients undergoing foot nail surgery. In addition, a questionnaire was used to collect other variables such as age, sex and educational level. The verbal numeric scale was employed to value the postoperative pain after 24 h. Age and sex influenced (*p* < 0.05) preoperative anxiety, which had a prevalence of 22.6%. More than 43% of patients needed more information and this was correlated with anxiety (r = 0.629; *p* < 0.001). There was a significant difference when comparing the total anxiety between the group of participants who had more pain and that who had less pain (*p* < 0.001). The prevalence of anxiety was high in the participants of this study, being greater in young patients and in women. There was a deficit of information, increasing the level of preoperative anxiety, which in turn was related with greater postoperative pain.

## 1. Introduction

Surgery is a stressful event that is frequently associated with anxiety in most patients. Preoperative anxiety is a response that is expected and is associated with emotional and physical problems [1,2]. Moreover, it can negatively influence both the intraoperative moment (for example, with a rise in blood pressure which can affect the surgery and increase complications [3]) and the postoperative moment, such as pain [4,5].

Surgeons may tend to underestimate the patients’ anxiety [6] and can disregard that the moment prior to surgery, in the waiting room, can be a stressful event for them. The level of anxiety and the etiology are diverse. Among the predictive factors, the complexity of the surgery, the anesthesia, the postoperative pain, not being appropriately informed and sociodemographic or psychosocial characteristics of the patient have been reported [7].

In the last decade the interest in studying the influence of preoperative anxiety on postoperative pain has increased [8,9,10]. It has also been demonstrated that high doses of anesthetics and postoperative analgesia are needed [4,11]. This can lead to a longer hospital stay, increasing the consumption of drugs and costs or the risk of readmittance, among others [12]. That is why it is important to assess and detect preoperative anxiety to help the patient and, avoiding it, impacts negatively all the surgical process. It would enable us, for example, including the patient’s emotional status in the preoperative assessment to treat it if necessary.

There is a great variety in the literature concerning the prevalence of preoperative anxiety recorded in different medical specialties. However, as far as we know, studies do not exist about the prevalence of anxiety in foot nail surgery, being the main type of surgery performed in Podiatry. The main aim of this study was to determine the prevalence of preoperative anxiety in foot nail surgery and the level of the patient’s demand for information. Secondarily, the study also aimed at checking whether or not preoperative anxiety was related to postoperative pain.

## 2. Materials and Methods

### 2.1. Setting

A cross-sectional study was done from January 2017 to February 2019, following the Strengthening the Reporting of Observational Studies in Epidemiology (STROBE) requirements. The participants were patients with foot nail surgery programmed in the Podiatric Clinical Area of the University of Seville. The Ethical Committee of the University Hospitals “Virgen Macarena” and “Virgen del Rocío”, (Seville, Spain) approved the study, with internal code 0859-N-18. All the patients signed a written consent.

### 2.2. Sample Size Calculation

Sample size was calculated using the formula:

$$n=\frac{N*Z_{\alpha }^{2}\;*p*q}{d^{2}*\left(N-1\right)+Z_{\alpha }^{2}*p*q}$$

*N* = Number of nail surgeries performed in the Podiatry Clinic Area of the University of Seville in the period estimated for data collection (3 years) = 251.

*Z* = 95% confidence level = 1.96

*p* = Estimated proportion = 28% (according to the average data reported in three studies: Laufenberg’s 2013 and 2015 and Kaydu & Gokcek’s 2019 [5,13,14]).

*d* = Precision = 5

The necessary recruitment number was *n* = 139. Finally, a total of 155 patients were recruited for the study.

### 2.3. Participants

The participants in this study were patients with foot nail surgery programmed in the Podiatry Clinical Area of the University of Seville. The nail surgery consisted of the partial or total ablation of the nail plate of the hallux, after nerve blocking with local anesthesia (Mepivacaine at 2%). Different procedures were employed according to the surgeon’s criterion. The inclusion criteria were to be over 18 years old, ASA I and II (American Society of Anesthesiologists), with an autonomous capacity to fill out the surveys and participate in the study. Patients with mental and/or emotional disorders, with cognitive deterioration, with incapacity to understand and respond to the questionnaires, those people medicated with anxiolytics before the surgery, with chronic pain in the foot and/or analgesic treatment for chronic pain, those who did not fulfill the postoperative recommendations and those with a contraindication to the study’s medical guideline were excluded from the study.

### 2.4. Data Collection

The data collection began in the immediate preoperative period; that is, in the waiting room, to register the moment of greatest anxiety as some authors indicate [15]. The participants were given a specific questionnaire and the Amsterdam Preoperative Anxiety and Information Scale (APAIS), which enables evaluating the preoperative anxiety of patients programmed for surgery, as well as the need for more surgical information [12,16]. The participants filled out the APAIS independently and without the presence of the researchers. Before the surgery a specific questionnaire was also used to collect other variables, which contained the sociodemographic and clinical data included in the literature as predictors of preoperative anxiety [8,14]. These were age, sex, educational level, previous experiences with surgery and anesthesia, and whether or not they felt nervous and why.

The APAIS scale has six statements: (1) I am worried about the anesthetic; (2) The anesthetic is on my mind continually; (3) I would like to know as much as possible about the anesthetic; (4) I am worried about the procedure; (5) The procedure is on my mind continually; and (6) I would like to know as much as possible about the procedure. These are divided into two subsections: One measures the patient’s anxiety related to the anesthesia (items 1 and 2) and the surgery (items 4 and 5). The other one measures the wish for more information about the anesthesia (item 3) and the surgery (item 6). The responses to the statements are evaluated with a Likert Scale (1-Not at all, to 5-Extremely).

The scoring of anxiety was obtained by summing the scores assigned to items 1, 2, 4 and 5. The sum of items 3 and 6 was calculated for the need of more information. The cutoff point established by the authors of the original version to determine clinical anxiety in a patient is ≥11 [16]. Furthermore, they recommend that with a score ≥5 the patients must receive more information about what they wish to be informed.

The day after surgery the maximum postoperative level of pain experienced in the first 24 postoperative hours was collected via the Numeric Rating Scale (NRS). This validated scale ranges from 0 (no pain) to 10 (extremely painful). For this study, as in previous studies [11], two standardized pain intensities were established: nothing-to-mild pain (NRS ≤ 3) and moderate-to-severe pain (NRS ≥ 4). This information was recorded by phone calls.

The same researcher did the explanation of the study to the participants and the data collection. The postoperative analgesic guideline was standardized in all the patients with 1 g of acetaminophen alternated with 600 mg ibuprofen every 8 h for the first day. The postoperative recommendations were also standardized: to keep the foot raised for 6–8 h, to walk 5 min every hour after the first 12 h, to avoid injury and direct exposure to sources of heat, not to remove or wet the bandage and to take the medication.

### 2.5. Statistical Analysis

The data analysis was done via the IBM^®^ SPSS Statistics statistical program version 25 for Windows 10 (IBM Corp, Armonk, New York, NY, USA).

The Cronbach α coefficient was calculated to evaluate the internal consistency of the two subscales. A Cronbach α > 0.70 was considered acceptable.

Descriptive analysis of the variables when they were of interest for the study (frequency, mean, standard deviation, median and interquartile range) was done. Normality tests were carried out (Kolmogorov–Smirnov when *N* > 50, and Shapiro–Wilk when N < 50), and comparisons between 2 groups were done via the Mann–Whitney U test and via the Kruskal–Wallis test or median test when 3 or more groups were compared, because data did not follow a normal distribution. Post hoc analysis was performed in case significant differences were obtained (Dunn’s test for Kruskal–Wallis, pair-wise comparisons via the Mann–Whitney U test for median test). The correlations were studied via Spearman’s rho test. Any value of *p* < 0.05 was considered significant.

## 3. Results

A total of 159 patients were initially recruited for the study. Four participants were excluded due to not fulfilling the postoperative indications. Finally, the statistical analysis was done with a total of 155 participants (122 women, 78.7%), average age 45.95 ± 21.44 years old. Other demographic characteristics are shown in Table 1.

Regarding the internal reliability of the two subsections of the APAIS scale, a Cronbach’s α of 0.82 was obtained for the items which measure anxiety and 0.73 for the items which measure the need for more information.

Table 2 shows the scores obtained in the APAIS anxiety subscale for all the participants, and by groups according to showing anxiety or not. Thirty-five participants (22.6%, 95% CI: 16%–29%) presented values corresponding to abnormal level of anxiety.

A moderate direct and significant correlation of r = 0.429 (*p* < 0.001) was found between the subsections which measure anxiety about anesthesia and anxiety about surgery. It was found that the greater the anxiety caused by the anesthesia, the greater the anxiety caused by the surgery.

Table 3 shows the distribution of the patients according to the demand for information.

Moderate or high level of information was needed by 43.9% of the participants. Among the 35 participants with anxiety, 40% of the patients had a moderate need for information and 20% had a high need for information. The need for more information was correlated with anxiety (r = 0.629, *p* < 0.001).

Table 4 shows the results of the analysis between the variables and the subscales of the APAIS, as well as the average anxiety scores obtained in each subgroup of the variable.

When preoperative anxiety was analyzed according to the different age groups using the Kruskal–Wallis test, a *p*-value equal to 0.001 was obtained. The Dunn’s test was then carried out to know the groups that showed the significant differences and a *p*-value equal to 0.003 was obtained between the “18 to 40 years old” and “41 to 65 years old” age groups and a *p*-value equal to 0.004 was obtained between the “18 to 40 years old” and “more than 65 years old” groups. A significant, although weak, inverse relation was also obtained between the age and the level of anxiety (rho = −0.326, *p* < 0.001) and between the age and the need for more information (rho = −0.252, *p* = 0.02).

The need for information was different according to the level of schooling of the participants (*p* = 0.003). The pair-wise comparisons revealed significant differences in the need for more information between those with a secondary and primary level of schooling (*p* = 0.025) and secondary and vocational training (*p* = 0.013).

No significant difference was found between the subscales of anxiety and need for information between men and women or between those who had prior experience with anesthesia or surgery and those who did not. However, among the patients with preoperative anxiety (i.e., more than 10 points in APAIS, N = 35), only 6 men were registered. Men reported a median (IQR) preoperative anxiety of 12 (1) and women 13 (5), and Mann–Whitney U test revealed a significant difference (*p* = 0.036). Furthermore, women with preoperative anxiety showed more need for information than men with preoperative anxiety (Median (IQR): 2(0), 6(4), *p* = 0.001)

The causes of anxiety referred by 62 patients were surgery (58.06%), previous experiences (16.13%), postoperative symptoms (9.68%), anesthesia (4.84%), fear of complications and lack of information (3.23% each) and other reasons (4.84%).

Table 5 reflects the anxiety scores according to the referred postsurgical pain groups.

A significant difference was obtained (Mann–Whitney U test, *p* < 0.001) when comparing the level of anxiety between the participants who had nothing-to-mild pain and those who had moderate-to-severe pain. That is, the greater the preoperative anxiety, the higher the postoperative pain. A weak, but significant relation was observed between both variables (Rho = 0.297, *p* < 0.001).

## 4. Discussion

This work aimed at evaluating preoperative anxiety in a very common surgical procedure in foot surgery - nail surgery. The APAIS score system was used, validated in Spanish and designed for elective surgery. It was used in other studies with acceptable reliability [12,13,14,16,17]. 

The evidence demonstrated that preoperative anxiety in this minor ambulatory surgery (foot nail surgery) was present in 22.6% of participants (35 out of 155). These results were similar to those obtained in other studies that used the APAIS, such as Kaydu and Gokcek [14] with patients undergoing extracorporeal lithiasis by shockwaves having a rate of 23.3%, Laufenberg et al. with a rate of 18.9% in elective surgery [13] or 42.7% in urological surgery [5]. Other authors reported between 38 and 80% [4,18,19,20,21]. These very high percentages could be due to the use of other scales, the type of anesthesia, the sample size or the place in which it was done. The surgical complexity is also noted as a risk factor of preoperative anxiety [22,23,24,25]. Mandy et al. highlighted the importance of evaluating preoperative anxiety in foot surgery [26]. These authors studied the evolution of the distinct affective states in 85 subjects undergoing different foot surgical procedures from the preoperative period until eight weeks after and obtained a moderate-high state of anxiety: 54%.

Preoperative anxiety was studied in several subgroups. Regarding to age, the younger patients had more preoperative anxiety than the patients over 65 years old. This coincides with previous works [21,27,28]. Bradshaw et al. [28] sustain that this could be due to the elderly having an easier acceptation of reality and probably having been exposed to the health system before. Other authors do not find this association [3,5]. Although preoperative anxiety was not different between men and women, in those participants with higher levels of preoperative anxiety (>10 points), it was found that women had greater anxiety than men, as previous studies also report [10,21,29]. A possible reason could be that women express their fears more freely [3]. This association was not found in a similar work that compared nail surgery anxiety by onychocryptosis (48 patients) with needle skin biopsy (50 patients) via the STAI scale [30]. Coinciding with Göktay et al. [30], the present study did not find that a low educational level impacted the anxiety as in other works [17,31]. Lanitis et al. indicated that having a lower educational level generates incapacity to efficiently handle the preoperative psychological state [32]. Regarding the anesthetic–surgical experience, Matthias et al. found that patients with prior experience were less anxious [17]. On the other hand, bad anesthetic–surgical experiences generate more anxiety concerning a future surgical operation [10,33], noted in this work as a cause of anxiety by 16.13% of participants. The main reason was the surgical procedure itself, experienced by 58.06% of participants. In the literature, these were the main motives along with surgical success, symptoms and general anesthesia [18,29].

A relevant finding was that the patients who had more postoperative pain had greater preoperative anxiety. This relationship has been previously observed in different medical disciplines, such as orthopedic knee and hip surgery [9,10], cesareans [34] and oral surgery [10]. In addition, opposite results can be found in the literature [5,23,26,35]. Borges et al. [34] indicated that the emotional status modulates pain, increasing the reactivity to pain and causing hyperalgesia.

In regard to the need for information, the results of the study showed that 43.9% of patients needed more information (>4 points in APAIS scale), this being greater in patients with anxiety. Perhaps surgeons underestimate the worries of the patients in simple surgical procedures, such as nail surgery. Ivette et al. [22] reported similar results with 40% of 100 patients for general surgery. On the other hand, Laufenberg et al. [13] noted this in 64% of 217 patients undergoing elective surgery. Ahmed et al. [35] reported that a high percentage of patients needed more information in spite of seeing an explanatory video about cataract surgery. The evidence demonstrates that preoperative visits and the availability of a person to inform the patient are more effective in diminishing anxiety than drugs [2,6]. Jawaid et al. [29] stated that 56% of the patients believed that their anxiety could have been less if they had received more detailed information. Further, we found that the patients with higher educational levels needed more information than those with lower levels, coinciding with other works [1,7,15]. This could be because they can express themselves better and are more aware of anesthesia and surgery [7]. Lastly, women needed more information than men when only patients with anxiety were analyzed. In addition, some authors reflect that patients without anesthetic–surgical experiences needed more information [1,7,33].

The findings of this study are novel because it is a type of surgery little studied before and they enable to know that there is high anxiety in a simple procedure, such as nail surgery. Furthermore, in the patients included in this study, anxiety influenced the postoperative pain. This could be taken into account to systematically register the level of preoperative anxiety and deal with it. More information should also be given to the patients. In addition, a new consultation could be facilitated when necessary in order to appropriately prepare the patient, increasing care quality and avoiding negative repercussions in the surgical process. The importance of evaluating and detecting preoperative anxiety is based on a high degree of anxiety being able to increase the complexity of the procedure [36]. This could diminish the patient’s compliance and comprehension of the instructions [10], which can affect the favorable development of the surgery and the care quality.

This study has certain limitations. One limitation could be the inclusion of more women than men, meaning that it was not possible to do more balanced comparisons. Nevertheless, the main variable (preoperative anxiety) was not different between men and women, as shown in Table 4. Another limitation may be having recruited the participants from one center; it prevents us from generalizing the results to other health centers. Finally, the same surgeon did not do all surgical procedures; data were collected from patients operated by six different surgeons. It could be another limitation, due to each surgeon could have transmitted different sense of security and tranquility during the consultations prior to surgery. However, it could also be interpreted as strength of this study, being advantageous to generalize the results. Another strength is related to the reliability of the APAIS subscales, as we got acceptable results very similar to those obtained in the original version [16], in the Spanish version [12] and by other authors [17].

Further research is needed to analyze the prevalence of anxiety in other types of foot surgery and determine if the information detailed influences the preoperative anxiety. Other studies could be oriented to checking the effectiveness of preoperative anxiolytic medication in comparison to alternatives to decrease anxiety.

## 5. Conclusions

In conclusion, it was observed that the prevalence of anxiety was high in participants of this study. Levels were greater in young patients and women. There was also a deficit of information, which was correlated with an increase in the level of preoperative anxiety. Furthermore, there was a relationship between preoperative anxiety and postoperative pain. Therefore, it is important to assess and detect the level of anxiety with appropriate instruments to treat it and improve the process of patient care.

## Figures and Tables

**Table 1:** Sociodemographic characteristics.

Variable	Group	*n* (%)
**Sex**	Men	33 (21.3%)
	Women	122 (78.7%)
**Age**	18 to 40 years old	61 (39.4%)
	41 to 65 years old	60 (38.7%)
	More than 65 years old	34 (21.9%)
**Level of Schooling**	None	11 (7.1%)
	Primary (6–12 years old)	25 (16.1%)
	Secondary (13–16 years old)	38 (24.5%)
	A-levels (17–18 years old)	16 (10.3%)
	Vocational Training	18 (11.6%)
	University	47 (30.3%)
**Anesthetized Before**	No	24 (15.5%)
	Yes	131 (84.5%)
**Operated Before**	No	44 (28.4%)
	Yes	111 (71.6%)

**Table 2:** Scores obtained in the Amsterdam preoperative anxiety and information scale (APAIS) anxiety subscale.

	Anxiety about Anesthesia Items 1 + 2 2–10 Points	Anxiety about Surgery Items 4 + 5 2–10 Points	Total Anxiety Items 1 + 2 + 4 + 5 4–20 Points
	Median (Interquartile Range)	Median (Interquartile Range)	Median (Interquartile Range)
All participants (*n* = 155)	3 (3)	4 (2)	7 (5)
Without anxiety (*n* = 120)	2 (2)	3 (1.75)	7 (3)
With anxiety (*n* = 35)	7 (3)	7 (3)	13 (4)

**Table 3:** Need for information according to APAIS in all the patients and those cataloged as with or without anxiety.

	Nothing or Little 2–4 Points	Moderate 5–7 Points	High 8–10 Points	Total Score Items 3 + 6 2–10 Points
	n (%)	n (%)	n (%)	Median (Interquartile Range)
All the patients (*n* = 155)	87 (56.1)	49 (31.6)	19 (12.3)	4 (4)
Without anxiety (*n* = 120)	73 (60.8)	35 (29.2)	12 (10.0)	4 (4)
With anxiety (*n* = 35)	14 (40.1)	14 (40.0)	7 (20.0)	5 (5)

**Table 4:** Results of the analysis between the variables and the subscales of the APAIS.

		Anxiety Items 1 + 2 + 4 + 5	Wish for more information Items 3 + 6
		Median (Interquartile Range)	Median (Interquartile Range)
Sex	Men	7 (5)	4 (3)
	Women	7 (5)	4 (2)
	*p* ^a^	0.092	0.162
Age	18 to 40 years old	9 (6)	4 (5)
	41 to 65 years old	7 (3)	5 (3)
	More than 65 years old	5.5 (6)	4 (3)
	*p* ^b^	0.001 **	0.097
Level of schooling	None	5 (10)	3 (4)
	Primary (6–12 years old)	8 (6)	5 (3)
	Secondary (13–16 years old)	7 (6)	3 (2)
	A-levels (17–18 years old)	6 (3)	4 (3)
	Vocational T.	9 (6)	6 (3)
	University	8 (3)	5 (5)
	*p* ^k^	0.259	0.003 *
Anesthetized before	No	6 (4)	3.5 (5)
	Yes	8 (5)	4 (4)
	*p* ^a^	0.314	0.324
Operated before	No	7 (4)	4 (4)
	Yes	7 (6)	4 (4)
	*p* ^a^	0.734	0.317

^a^ Mann–Whitney U test; ^b^ Kruskal–Wallis test; ^k^ Median test; * *p* < 0.05; ** *p* < 0.01.

**Table 5:** Postoperative pain after 24 h according to the APAIS scores. Anxiety was dichotomized according to the cutoff point established by the authors of the original version of APAIS to determine clinical anxiety (≥11) [16].

		Anxiety APAIS 1 + 2 + 4 + 5	*p*-Value
Pain	*n* (%)	Median (Interquartile Range)	
Nothing to mild (NRS ≤ 3)	89/155 (57.4%)	7 (4)	<0.001
Moderate to severe (NRS ≥ 4)	66/155 (42.6%)	9 (7)	
